# Monoallelic Chromatin Conformation Flanking Long-Range Silenced Domains in Cancer-Derived and Normal Cells

**DOI:** 10.1371/journal.pone.0063190

**Published:** 2013-05-16

**Authors:** Domenic Di Paola, John Raelson, Emmanouil Rampakakis, Mark Basik, Maria Zannis-Hadjopoulos, W. Edward C. Bradley

**Affiliations:** 1 Goodman Cancer Centre, McGill University, Montreal, Quebec, Canada; 2 Genizon Biosciences, St. Laurent, Quebec, Canada; 3 Department of Biochemistry, McGill University, Montreal, Quebec, Canada; 4 Department of Oncology, McGill University, Montreal, Quebec, Canada; 5 Department of Medicine, Université de Montreal, Montreal, Quebec, Canada; Florida State University, United States of America

## Abstract

Epigenetic inactivation of chromatin plays an important role in determining cell phenotype in both normal and cancer cells, but our knowledge is still incomplete with respect to any potential monoallelic nature of the phenomenon. We have genotyped DNA isolated from chromatin of two colorectal cancer-derived lines and a culture of normal human intestinal epithelial cells (HIEC), which was immunoprecipitated with antibodies to acetylated vs. methylated histone H3K9, and presented the data as B allele frequency differences over multiple single-nucleotide polymorphism (SNP) moving window averages. [B allele is an arbitrary term defined as one of the two alleles at any given SNP, named A and B]. Three different validation tests confirmed that peaks exhibiting differences represented monoallelic domains. These complementary tests confirmed the following: 1) genes in the regions of high B allele frequency difference were expressed monoallelically; 2) in normal cells all five imprinting control regions which carried heterozygous SNPs were characterized by B allele difference peaks; and 3) the haplotypes in the B allele difference peaks were faithfully maintained in the chromatin immunoprecipitated with the respective antibodies. In both samples most of the monoallelic domains were found at the boundaries between regions of open and closed chromatin. With respect to the cancer line, this supports the established concept of conformation spreading, but the results from the normal cells were unexpected. Since these cells were polyclonal, the monoallelic structures were probably not determined by random choice as occurs in X-inactivation, so we propose that epigenetic inactivation in some domains may be heritable and polymorphic in normal human cells.

## Introduction

Epigenetic control of gene expression is an important source of phenotypic variation in cancer. At the chromosomal level, this control is manifested by alterations in chromatin conformation associated with histone modifications, such as methylation and/or acetylation of lysine residues in N-terminal tails of histones, and with methylation of C residues in CpG-rich islands in or near gene promoters. These alterations have been used extensively as markers to determine the epigenetic status of a given gene, and recent advances in genome technology have allowed assessment of epigenetic inactivation over megabase ranges; it is now thought that these changes may spread across long stretches [1]. Our knowledge in this area is nevertheless incomplete for several reasons: first, CpG island methylation only yields details of individual genes which are associated with CpG islands, and this only in cases where gene inactivation genuinely corresponds with methylation status. Second, although these recent studies have indeed demonstrated that large-scale epigenetic changes may occur, few exhaustive genome-wide analyses comparing normal and cancer-derived cells have been undertaken.

Another aspect of this phenomenon which has remained relatively unexplored is the degree to which epigenetic inactivation may be restricted to one allele. Within normal cells, allele-specific gene inactivation is known to occur at a number of loci, including genes within imprinted domains (where the choice of which allele is inactivated is dependent on the parent of origin), most genes on one X-chromosome in female cells, odorant receptors, and at a number of genes involved in inflammation and the immune response (reviewed by [2]). Control mechanisms for some of these inactivation events appear to be altered in cancer cells, such that, for example, imprinted loci are often biallelically expressed in cancer (reviewed in [3]). However, the extent to which de novo monoallelic inactivation occurs in cancer cells is unknown. Early work by us [4], [5] and by others [6] indicated that in the model mammalian cell line CHO, monoallelic inactivation may spread over a megabase range such that selection against expression of a single allele at one locus resulted in suppression of one allele at a second, linked locus. Such spreading was shown to occur at high frequency and did not involve methylation of the promoter-associated CpG island for at least one of the genes involved [7]. More recently, we have shown that in about one-half of tumor-derived cell lines in which RARB, a gene with tumor-suppressive effect, was completely inactivated, the promoter of only one of the two alleles was methylated [8], again suggesting a methylation-independent inactivation mechanism, which may be monoallelic in nature.

In this study, we used antisera against histone H3 acetylated at K9/14 (H3Ac) and histone H3 tri-methylated at K9 (H3M) in ChIP experiments on two colorectal cancer-derived lines (HCT116 and Colo205) and a culture of human intestinal epithelial cells (HIEC), which are non-immortalized and polyclonal. These histone marks are associated with open and closed chromatin conformation, respectively, and were selected from among several known to be associated with conformation in part because the modifications are mutually exclusive and there may be a greater chance that the data would be unequivocal. This also simplified the determination of allelic bias in conformation at each SNP used to genotype the DNA extracted from the immunoprecipitated chromatin, since we could calculate the difference in B allele frequencies determined at each SNP of the HAP550k microarrays. Not unexpectedly, long ‘gene deserts’ were found to be characterized by stretches of generally closed chromatin in both normal and cancer cells. Of interest, we found that monoallelic conformation was preferentially localized at the boundaries of these long stretches of closed chromatin conformation, suggesting spreading of the inactive (or active) conformation.

## Results and Discussion

**Figure 1:**
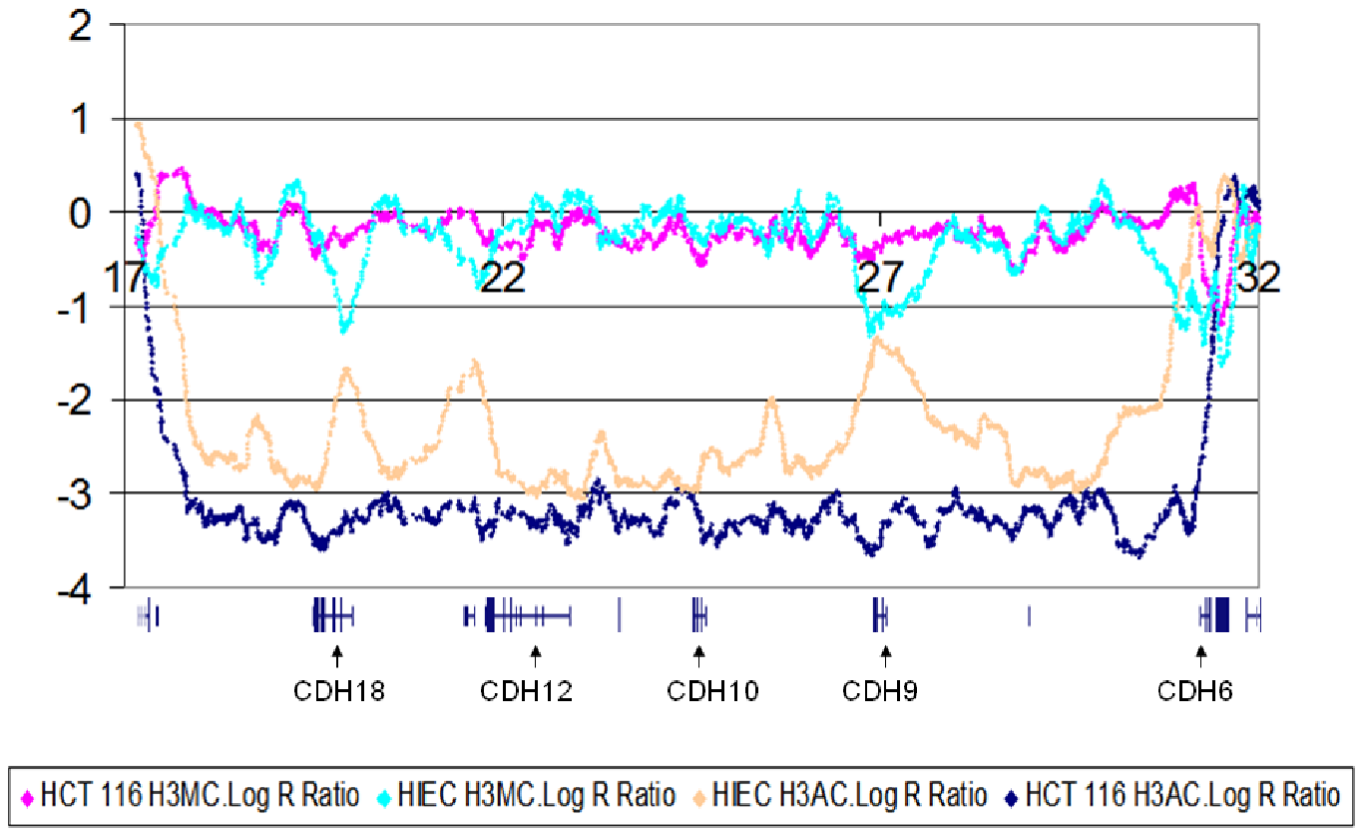
LogR ratios (fluorescence intensity) of chromatin immunoprecipitated (ChIP) with anti-H3K9 acetylated or anti-H3K9 trimethylated antisera showing chromosome 5, 17–32 Mb; this is a gene-poor domain. Plotted values represent the average readings of a 21-SNP moving window. HCT116, a colorectal cancer-derived cell line; HIEC, normal human intestinal epithelial cells. Genes (from UCSC Genome Browser, hg18) are depicted below the LogR plots.

To generate long-range images of chromatin conformation, the fluorescence intensity values, expressed as LogR ratios, were averaged over moving windows comprising 21 or more SNPs, and plotted against chromosomal position. As expected, based on data provided in the UCSC epigenome browser, gene-poor genomic regions were characterized by stretches of low LogR for anti-H3Ac-precipitated chromatin and correspondingly high LogR for anti-H3M. This is illustrated in Fig. 1, showing a 15****Mb domain of Chr5 harbouring fewer than 10 genes, five of which are members of the cadherin family. In the normal cells (and less often, in the cancer cell lines) these regions were typically interrupted by small islands of open conformation marked by peaks of the anti-H3Ac plot, mirrored by troughs of the anti-H3M plot. Sometimes but not always, these corresponded to the promoters of the rare genes in the domain (Fig. 1, *CDH18* and *CDH9* and the uncharacterized gene fragment at 21.5****Mb, downstream of *CDH12*). The peaks in these domains were often flattened in the cancer-derived lines, and the plot presented in Fig. 1 suggests that shifting of the right-hand boundary may have occurred such that *CDH6* is in closed chromatin in HCT116 cells. It is possible that this apparent shifting is at least sometimes part of the process of tumour progression (see below).

**Figure 2:**
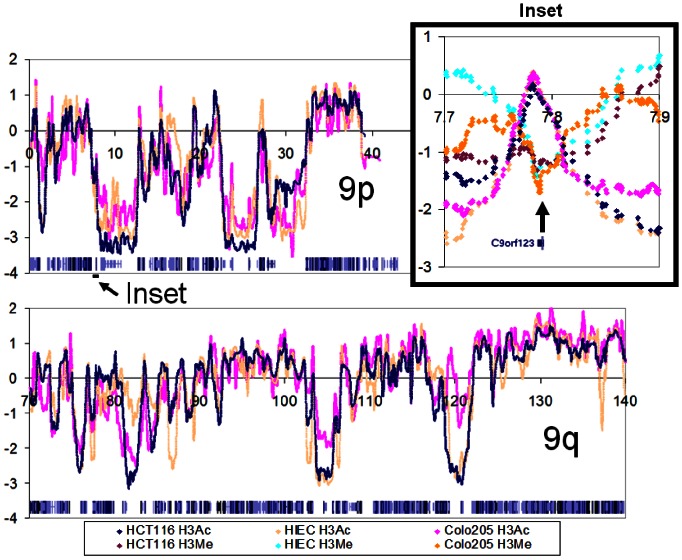
LogR values of ChIP material showing all of chromosome 9 for HIEC, HCT116 and a second colorectal cancer cell line, Colo205. Data were treated as described in the legend to Fig. 1. Inset, the region between 7.7****Mb and 7.9****Mb, showing the peaks of ChIP using anti-acetylated antiserum vs. the troughs of ChIP using anti-methylated serum, coinciding with the promoter region of C9orf123. LogR plots of anti-methylated ChIP are omitted from the main figure for clarity.

The patterns of LogR ratios for the whole of chr9 are shown in Fig. 2, with UCSC Genome Browser ‘dense’ display of genes at the bottom of each graph segment. Again, gene-poor domains (for example, those centered around 10****Mb, 82****Mb, 105****Mb) are characterized by low LogR values of anti-H3Ac interrupted by localized peaks which are occasionally flattened in the cancer lines (H3M data are not included in the main part of the figure for simplicity). In the inset of Fig. 2 is displayed a peak in Chr9 for all three of the anti-acetylated ChIP samples with a concomitant trough for all three of the anti-methylated ChIPs, consistent with our expectation of mirror-images in the LogR plots of the two immunoprecipitated samples. There is only one gene in the region, C9orf123, whose 5′end is very close to the respective peak and trough.

**Figure 3:**
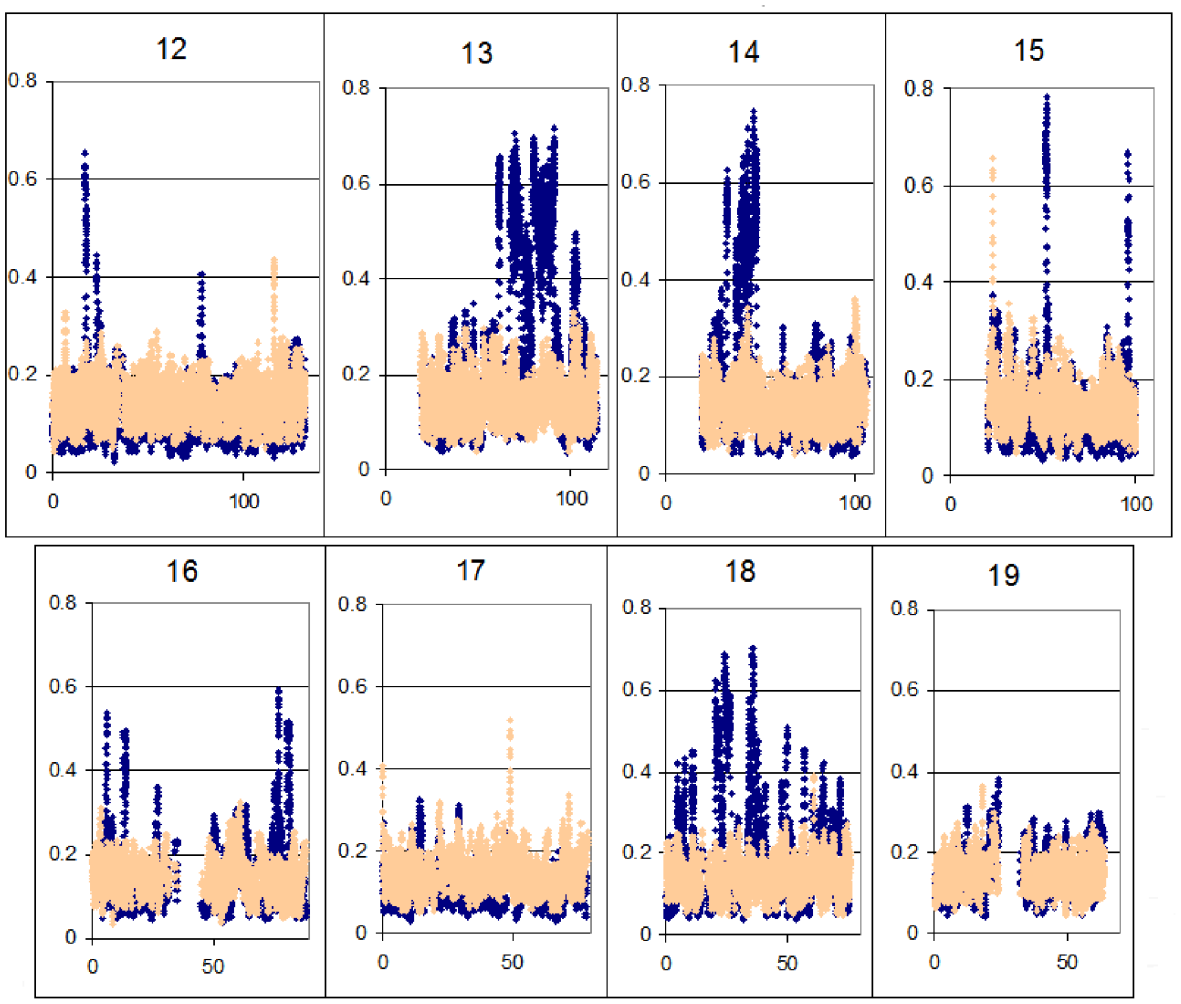
B-allele frequency (BAF) differences for chromosomes 12–19. As described in the text, for each heterozygous SNP absolute values of BAF differences between the two ChIP samples were calculated and 11-marker moving window averages across each chromosome were plotted. Blue, HCT116; coral, HIEC. Genes (UCSC Genome Browser) are depicted below the LogR plots.

We then determined the patterns of allelic bias in chromatin conformation for the two near-diploid cell samples (HCT116 and HIEC), expressed as B allele frequency (BAF) difference between the two ChIP samples averaged over 11-marker moving windows (see Methods). We found more than 60 peaks above our conservative cut-off of 0.35 (see text in File S1) which extended for as much as 10****Mb at a stretch in the cancer-derived line HCT116. About 35 such peaks were observed in the HIEC cells, the widest covering a range of about 400****kb (width of the peak at one-half height; see Fig. 3 for overviews of several chromosomes).

To validate these data we performed both structural and functional tests (details in Methods). First, over the length of a BAF difference peak a relatively closed conformation should extend uninterrupted on one homologue and an open conformation on the other. This was shown to be the case over runs of SNPs in high linkage disequilibrium (LD), as we found perfect concordance between haplotype phase and the sequence of alleles enriched in the respective ChIP samples (binomial p = 1.4×10^−45^ for HCT116, 5.8×10^−11^ for HIEC; data in Figs. S1 and S2 in File S1). Second, we found that seven of seven genes residing in peaks of BAF difference were monoallelically expressed (Fig. S3 in File S1). Third, we showed that five peaks of BAF difference in HIEC, including the major peak at 22.9****Mb of chromosome 15 (Fig. 3), corresponded precisely with imprinting control regions (ICRs), the short monoallelically methylated DNA sequences which direct imprinting (other data not shown). (Other ICRs we queried were homozygous in HIEC). Overall, the three tests we performed make a compelling case that BAF differences reflect the authentic allelic differences in conformational structure of the chromatin.

**Figure 4:**
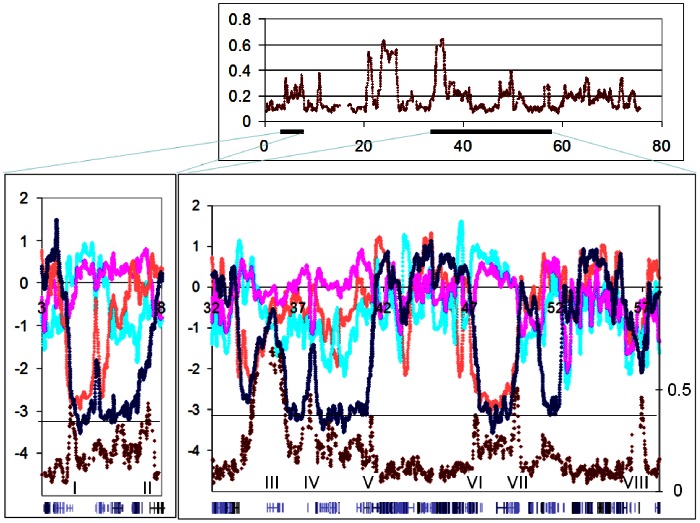
Domains of monoallelic conformation are found at boundaries of open/closed conformation in HCT116 cells. Upper panel, BAF differences across the whole of chromosome 18 for HCT116. Lower panels, plot of HCT BAF difference (Red; right hand scale) superimposed on plots of HCT and HIEC, LogR ratios (left hand scale) over the chromosome 18 domains indicated in the upper panel.

When the LogR ratios and BAF differences of HCT116 cells were plotted together two general patterns were observed (see Fig. 4 which shows eight of the peaks on chromosome 18, numbered I-VIII). As might be expected, some BAF difference peaks corresponded closely with isolated peaks and/or troughs of the respective ChIP samples. Those at 35****Mb and 57****Mb (peaks III and VIII) fall into this category, along with about one-quarter of the rest of these peaks throughout the genome. As many as two-thirds of the peaks, however, occurred at the boundaries between low and high LogR values, that is, between domains of open and closed chromatin conformation. Peaks I, II, V – VII and perhaps IV are in this category (Fig. 4; the last is less certain because of uninformative homozygosity straddling the peak). In most cases the LogR and BAF difference plots were virtually superimposable over the immediate region of interest, indicating that divergence between the alleles began at the same point as the beginning of the switch from one conformation to the other. Intriguingly, the boundaries coinciding with BAF difference peaks were usually those for which a relative displacement was seen in LogR ratios, as is seen for peaks I, VI and VII (the last is smaller, but real, with a displacement of 100****kb). The boundary at peak II can also be considered as displaced, by a longer distance of about 2****Mb.

We interpret this as indicating that either open or closed domains have spread at these boundaries and that the spread was either monoallelic or occurred to different extents on the two homologues, thereby generating a domain of monoallelic chromatin conformation. Alternative explanations are possible, but we favor the spreading scenario in part because it seems simple to envision such a mechanism, for which some precedent exists in mammalian cells in the form of X-inactivation spreading into autosomal chromatin at the fusion points of X-autosome translocation [9].

**Figure 5:**
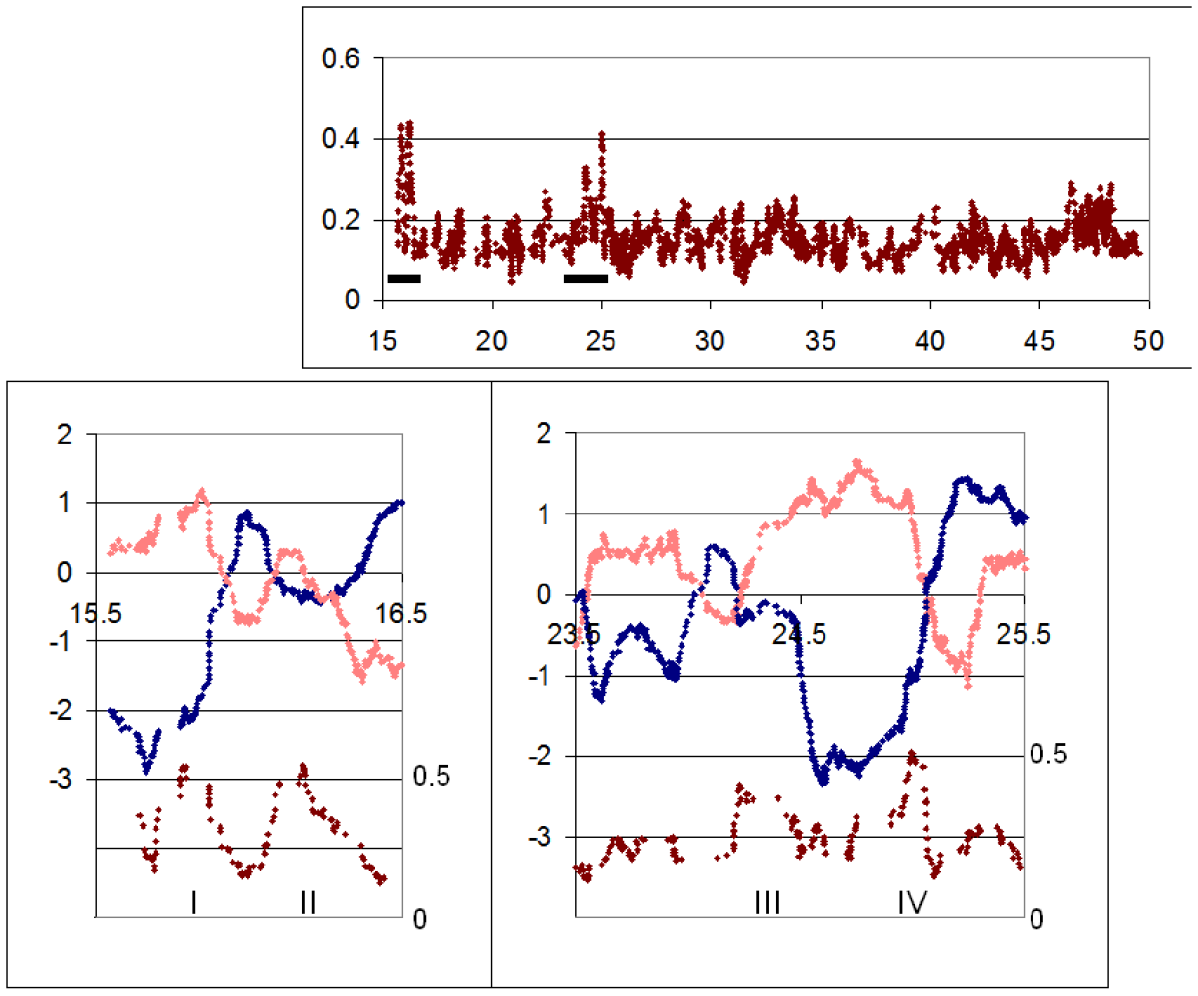
Domains of monoallelic conformation are found at boundaries of open/closed conformation in HIEC cells. Upper panel, BAF differences across the whole of chromosome 22 for HIEC. Lower panels, HIEC BAF difference plot (Red; right hand scale) superimposed on HCT and HIEC, LogR ratios plots (left hand scale) over the chromosome 22 domains indicated in the upper panel.

We also performed the same analyses with data from HIEC cells. The X chromosome yielded no BAF differences above the background noise (data not shown), even though H3K9 acetylation/methylation is involved in X-inactivation [10]. This is as expected for random inactivation, considering that HIEC cells are polyclonal, and the result confirmed that random inactivation events should not confound our results. Among the autosomes, we judged 13/32 BAF difference peaks as corresponding to isolated LogR ratio peaks/troughs of H3Ac/H3M ChIP samples. However the rest, comprising a majority of the BAF difference peaks coincided with boundaries between open and closed chromatin conformation (examples shown for chromosome 22 in Fig. 5 with peak II being in the former category and the others showing monoallelic boundary shift effects). This was an unexpected result, which we nevertheless consider to be real, given the rigor of our validation process. Our interpretation of this effect is as described above, namely that at boundaries of open/closed chromatin conformation, some degree of monoallelic spreading of conformation may occur.

To what can we attribute this apparent spreading? The polyclonal nature of HIEC allows us to rule out some possibilities. First, it is unlikely to be due to the same mechanism as the widespread monoallelic expression described by Gimelbrant et al. [11], since the latter was shown to be random. We can similarly rule out the possibility that the peaks reflect a precancerous state, as is seen with methylation of tumor suppressor genes in normal tissue of cancer patients, since this would also be random with respect to choice of allele. In any event, the HIEC cells are fetal in origin and not immortalized [12], and would, therefore, not be expected to have any precancerous characteristics. Another interpretation, which, however, is not strongly supported by the data, is that these peaks of BAF difference are previously unknown imprinted domains. Recent evidence from genome-wide searches suggests that most genuine imprinted domains are now known, so given their number (approximately 20) and their presence at open-closed chromatin boundaries (unlike the imprinting-associated peaks that we did detect) this seems unlikely, although this issue cannot be unambiguously addressed using our experimental design. One interpretation we consider to be consistent with the data is that the allelic differences detected reflect constitutional epigenetic changes, i.e., that the monoallelic peaks would be present in all tissues of the individual in question. Such phenomena have been described for a limited number of tumor suppressor genes, including *MGMT* [13], in that these genes have been found to be silenced in normal somatic cells, and are thought to result in a cancer-predisposing phenotype. There are suggestions in the literature that these ‘constitutional epimutations’ may be genetic in origin [14], [15]. Other groups have performed genome-wide association studies of allelic expression [16] and have found many SNPs to be associated with expression levels. In all these cases, the monoallelic effect is restricted to one genetic component, such as a SNP or the promoter of a single gene. There was no particular association in these studies of monoallelic expression with chromatin conformation or with proximity to gene deserts, so it is still unknown whether the monoallelic expression patterns these authors describe may overlap with those that we describe.

Our study breaks new ground in two respects. First, it localizes a significant source of monoallelic chromatin structure to regions immediately adjacent to domains of closed chromatin conformation. As such, it suggests a mechanism for this epigenetic polymorphism, which may resemble a controlled version of the spreading that we and others propose to be a significant source of phenotypic variation in cancer. Second, if our interpretation is accurate, it raises the prospect of a genetic component to this source of epigenetic variation. It is known that imprinting at the *IGF2R* site on chr6q is polymorphic, and perhaps some elements of heritable epigenetic control of chromatin conformation may exist, which manifest themselves as the domains of monoallelic conformation that we have documented. An unexplained phenomenon, which may be related is transgenerational inheritance [17], in which genetic factors influence phenotype in successive generations without the genes directly determining that phenotype being inherited. In any event, the novel aspects of our results may lead to a deeper understanding of heredity, and we are currently investigating these effects further.

## Methods

### Cells

HIEC-6 are polyclonal non-immortalized intestinal epithelial cells cultured from a female 20-week embryo. They were kindly furnished at passage 17 by Jean-François Beaulieu, and were grown according to published methods [12]. Experiments were performed within 6 passages of reception in our laboratory. HCT116 and Colo205 are colorectal cancer-derived cell lines originally obtained from ATCC, and grown in RPMI as previously described.

### Chromatin Immunoprecipitation (ChIP)

The procedure followed was that described by [18]. Cells cultured in complete media were washed with pre-warmed PBS and treated with 1% formaldehyde for 10 min to cross-link proteins and DNA *in vivo*; they were then washed and scraped into ice-cold PBS and resuspended in lysis buffer (50 mM HEPES-KOH pH 7.5, 140 mM NaCl, 1% Triton X-100, 2 mM EDTA) supplemented with a complete protease inhibitor tablet (Roche Molecular Biochemicals). Following passage through a 21-G needle three times, the nuclei were harvested, resuspended in one packed nuclear volume of lysis buffer, and sonicated until DNA fragments of <1 kb were obtained. Chromatin size was monitored by electrophoresis. Immunoprecipitation (IP) was carried out as previously described, with the following modifications: Briefly, sheared chromatin lysates (500 µg) were pre-cleared by incubation with 50 µl of protein A/G agarose (Roche Molecular Biochemicals) to reduce background caused by non-specific adsorption to the beads, incubated for 6 h with either 20 µg of anti-acetylated H3K9/14 (Upstate), anti-trimethylated H3K9 (Upstate) or normal rabbit serum (NRS) at 4°C with constant rotation. Protein A/G agarose (50 µl) was added and incubated overnight at 4°C. The pelleted beads were washed successively twice with 1 ml of lysis buffer for 15 min each at 4°C, followed by 1 ml of WB1 (50 mM Tris–HCl pH 7.5, 500 mM NaCl, 0.1% NP40, 0.05% sodium deoxycholate, complete protease inhibitor tablet), 1 ml of WB2 (50 mM Tris–HCl pH 7.5, 0.1% NP40, 0.05% sodium deoxycholate, complete protease inhibitor tablet) and 1 ml of sterile TE. The beads were resuspended in 200 µl TE/1% SDS, incubated at room temperature (rt) for 15 min and centrifuged at 3000 r.p.m. for 1 min at rt. Half of the supernatant was then incubated overnight at 65°C to reverse the cross-links, followed by 100 µg of proteinase K at 55°C for 2 h. The DNA was purified using QIAquick PCR purification kit (Qiagen, Valencia, CA, USA) and eluted in 100 µl TE.

### Genotyping

DNA from HIEC and HCT116 was genotyped on the Illumina HAP550 microarrays by the genotyping service of Genizon Biosciences Inc., according to the manufacturer’s instructions and as has been described [19]. DNA from Colo205 was genotyped on the 1****M duo array, according to the manufacturer’s instructions. To account for the difference in microarray manufacture, we multiplied the LogR ratios from the 1****M array by 2.0 for presentation with those plots generated from the HAP550 array. This allowed presentation of plots with equivalent amplitude. Microarray data are deposited in Dryad (doi:10.5061/dryad.43h8c).

### Calculating B Allele Frequency Differences

We plotted B-allele frequency (BAF) differences against chromosomal position, presenting the data in two ways. First we calculated the deviation from the BAF of the control chromatin preparation precipitated with normal rabbit serum for each of the two immunoprecipitated samples. The difference between these values was calculated at each SNP, the absolute value was derived and 11-marker moving window averages were plotted vs. chromosomal position. Second, for each SNP we simply subtracted BAF for the H3Ac sample from that for the H3M sample (normalized) and the absolute values of these differences were averaged over 11-marker moving windows as above and plotted vs. position. There was essentially no difference between the two methods of calculation, and we present only data derived by the second of the two methods. Three tests were performed to validate the results, as described in the following section.

### Validation of BAF Difference Peaks as Representing Allelic Bias

To validate these data we performed both structural and functional tests. First, if peaks of BAF difference reflect actual conformational allelic bias, then an open conformation should extend over the length of BAF difference on one homologue and closed conformation on the other. Thus, patterns of BAF differences in the respective ChIP samples should appear as haplotypes of one or the other homologue. We found the patterns to be perfectly consistent (binomial p = 1.4×10^−45^ for HCT116, 5.8×10^−11^ for HIEC; data for several LD blocks within two peaks are given in File S1, Fig 2, Fig. 3). The second validation test was based on the expectation that within a peak of BAF difference, only one of the alleles carried a gene in open conformation, so the expression pattern of this gene should tend to be monoallelic. Samples of cDNA of seven genes in these regions which carried heterozygous cSNPs were assayed and found to have significant or complete enrichment for one allele (File S1, Fig. S3). The third test was based on the expectation that at least some of the BAF difference peaks in HIEC cells should correspond to known domains of monoallelic expression in normal cells. Since HIEC are polyclonal, we should not detect any allelic bias on the X chromosome or at other loci subject to random allelic inactivation (File S1); however, in imprinted domains, allelic inactivation is not random but parent-specific, so all cells in a polyclonal population should carry the imprinting mark on the same allele. Accordingly, we found five of the HIEC BAF difference peaks, including the major peak at 22.9****Mb of chromosome 15 (Fig. 3), corresponded precisely with ICRs (File S1). All the other ICRs we queried, including that controlling the H19 cluster, are homozygous in HIEC and therefore undetectable.

## Supporting Information

File S1 **Supporting Text and Figures S1, S2 and S3. Figure S1. Validation that BAF difference peaks reflect contiguous domains in HCT116.** We reasoned that if BAF differences represented authentic differences between the two homologs, the material immunoprecitated by anti-Me should mainly be derived from one homolog, and that by anti-Ac mainly from the other throughout the length of high LD. Two regions under the long peak of BAF difference on chr1 (upper panel) were found for which HapMap data showed very high LD (r^2^ more than 0.9 throughout). The major allele frequency was plotted for anti-Ac (blue) and anti-Me (coral) for each SNP within the identified run of high LD. For every SNP, the anti-Ac ChIP yielded a higher BAF than the anti-Me ChIP, indicating perfect concordance between haplotype and immunoprecipitated material. **Figure S2.** **Validation that BAF difference peaks reflect contiguous domains in HIEC.** See legend to Figure S1. Three regions of high LD on chr7 are shown. Again perfect concordance was observed between haplotype and immunoprecipitated material. **Figure S3. Validation that BAF difference peaks represent domains of monoallelic expression at SPRY2 (at 80.1 Mb, chr13).** Genomic DNA and cDNA around rs504122 (C/T heterozygous in both HCT116 and HIEC) were amplified from both lines and sequenced. Upper panels, both alleles are expressed in HIEC, only the C in HCT116. Lower panel, BAF differences plotted for both cell lines, showing HCT116, but not HIEC, with BAF differences.
(DOC)
