# Supplementary material for: Monoallelic Chromatin Conformation Flanking Long-Range Silenced Domains in Cancer-Derived and Normal Cells
Source: PLoS One. 2013 May 16;8(5):e63190. doi: 10.1371/journal.pone.0063190 (PMC3655995; doi:10.1371/journal.pone.0063190)
Supplement: File S1 — Supporting Text and Figures S1, S2 and S3. Figure S1. Validation that BAF difference peaks reflect contiguous domains in HCT116. We reasoned that if BAF differences represented authentic differences between the two homologs, the material immunoprecitated by anti-Me should mainly be derived from one homolog, and that by anti-Ac mainly from the other throughout the length of high LD. Two regions under the long peak of BAF difference on chr1 (upper panel) were found for which HapMap data showed very high LD (r2 more than 0.9 throughout). The major allele frequency was plotted for anti-Ac (blue) and anti-Me (coral) for each SNP within the identified run of high LD. For every SNP, the anti-Ac ChIP yielded a higher BAF than the anti-Me ChIP, indicating perfect concordance between haplotype and immunoprecipitated material. Figure S2. Validation that BAF difference peaks reflect contiguous domains in HIEC. See legend to Figure S1. Three regions of high LD on chr7 are shown. Again perfect concordance was observed between haplotype and immunoprecipitated material. Figure S3. Validation that BAF difference peaks represent domains of monoallelic expression at SPRY2 (at 80.1 Mb, chr13). Genomic DNA and cDNA around rs504122 (C/T heterozygous in both HCT116 and HIEC) were amplified from both lines and sequenced. Upper panels, both alleles are expressed in HIEC, only the C in HCT116. Lower panel, BAF differences plotted for both cell lines, showing HCT116, but not HIEC, with BAF differences. (DOC) [file pone.0063190.s001.doc]

**Supporting information**

**Characteristics of the anti-H3Ac and anti-H3M plots**

1. General.

The rationale for choosing these two antibodies for ChIP was in part based on the expectation that the plots would reveal complementarity, in the sense that at H3K9 the chromatin would be either acetylated or methylated, generating peaks and troughs which mirror each other. If this were found, our confidence in the results would be enhanced. As seen in the figures of chromosome-wide plots (e.g., main text figs. 1, 2, 4, 5), this was indeed found, especially for closed domains of low acetylated (Ac) and high methylated (Me). Because of the log scale, Me troughs of relatively low apparent magnitude usually corresponded with peaks of Ac of greater magnitude (e.g., features at 21.5Mb and 25.5 Mb, Fig. 1, main text). Due to the expected noise, the Me troughs were sometimes not visible at all.

2. BAF bias at varying LogR ratio values.

Ge et al. (2009; main text reference) showed that average B allele frequency drifted lower with increasing R values in the 1M microarray from Illumina. We used a different platform (the 550K array), but we nevertheless performed the same exercise as described in Supplementary Figure 2 of Ge et al., and also found a similar skewing of BAF with increasing LogR (data not shown). Upon application of a correction algorithm similar to that described, however, we found no noticeable effect on the plots compared with the uncorrected results. This is possibly because we only considered highly significant BAF difference peaks in our analyses in contrast to Ge et al. who depended on BAF differences as little as 0.05.

3. Multiple-marker moving window averages.

To determine the long-range conformational tendencies we averaged BAF differences over 11 or 21 markers (for chromosome-wide plots, such as Figures 4 and 5, main text), and LogR ratios usually over 21 markers. The mathematical trade-off is the higher the number of markers, the greater the loss of detail, especially in narrow peaks and troughs. For certain discussion points we present averages over smaller moving windows, and occasionally no averaging at all.

**Calculating B allele frequency differences**

We plotted B-allele frequency (BAF) differences against chromosomal position. For each SNP we subtracted BAF for the H3Ac sample from that for the H3M sample (normalized) and the absolute values of these differences were averaged over 11-marker moving windows as above and plotted vs. position. This gave several peaks per chromosome which we judged to be robust, since these same domains showed deviations from 0.5 BAF, in the opposite direction, in both ChIP samples when they were analyzed separately. We conclude that these were domains with different conformation on the two homologues. Three tests were performed to validate this conclusion (next section).

**Validation of BAF difference peaks as representing allelic bias**

The availability of data indicating the presence of long domains of significant BAF differences presented options for validating these results with both structural and functional tests. First, we reasoned that if the BAF difference peaks reflected genuine differences in conformation, the open conformation should extend linearly over the entire domain of BAF difference on the same homologue; thus runs of heterozygous SNPs within such a domain which are in strong linkage disequilibrium (LD) should maintain the same phase in both haplotype analysis and the ChIP genotypes. For each of HCT116 and HIEC, we chose four BAF difference peaks on different chromosomes for analysis. For the reference SNP of each LD cluster (r^2^≥0.9 for HCT; ≥0.8 for HIEC), the major allele (A or B) was determined from the database of the genotyping service provider (Genizon Biosciences Inc.) and major allele frequency for each of the immunoprecipitated samples was determined either by taking the BAF directly, when the reference SNP major allele was B, or calculating 1-BAF when the major allele was A. For each SNP in high LD with the reference SNP the process was repeated and the data for the longest LD blocks are presented as scatter graphs of major allele frequency in the two ChIP samples (SI Fig. 1, 2). We found in every pair-wise comparison between SNPs in high LD phase was maintained perfectly. In total, 149 comparisons yielded consistency in the HCT dataset (binomial *P* = 1.4x10^-45^) whereas 34 concordances were found in the HIEC peaks (*P* =5.8x10^-11^). A single case was found for which the genotyping presumably failed, as both BAF determinations were near zero.

We then performed a second validation test on the HCT116 dataset, which was based on the expectation that within a peak of BAF difference, only one of the alleles carried a gene in open conformation so the expression pattern of this gene should be monoallelic. A number of genes were found in these regions which carried cSNPs for which the HCT line was heterozygous at the genomic level. The cDNA was amplified and sequenced and in each case there was relative enrichment for the allele on the homologue which was immunoprecipitated with anti-H3Ac (SI Figure 3). In addition, we twice genotyped the cDNA isolated from HCT116 and the data were concordant for the two genes in this group which were evaluable. It is of interest that two of the genes which we found to be monoallelically expressed were associated with peaks of BAF difference which were extremely narrow, affecting only the promoter of the gene in question. This indicates that a gene does not have to be entirely or even substantially within a domain of closed conformation to be inactivated, as long as the 5’ end of the gene is in this region.

Finally, we expected that at least some of the BAF difference peaks in HIEC cells should correspond to known domains of monoallelic expression in normal cells. Since HIEC are polyclonal, we would not expect to detect either X-associated allelic bias or genes subject to random allelic inactivation such as odorant receptors, but imprinted domains, or more specifically, their respective imprinting control regions (ICR), may be detectable. Five of the 25 peaks clearly corresponded to these regions. The SNP at the peak at Chr15, 22.9Mb (main text Fig. 3) is within 20kb of the associated CpG island, and the overall peak encompasses the whole island. Similarly, a cluster of 5 consecutive heterozygous SNPs with average BAF of 0.5 corresponds exactly to KCNQ1OT1, the antisense RNA gene which has been identified as the ICR for one segment of the extended imprinted domain at 11p15.5. Third, a double peak at chr14, 100.347Mb and 100.399Mb corresponds very closely with the differentially methylated CpG islands associated with the two ICRs controlling imprinting at the DLK1-MEG3-RTL1 cluster. Fourth, the centre of the ICR controlling imprinting at 6q24 is at the 5’ end of HYMAI (144,371,560), an untranslated gene starting at the second exon of PLAGL1 (which may also be used as an alternative TSS for PLAGL1). The only SNP in the CG island has a BAF difference of 0.764 which is 5.6 SD from the mean, *P*<10^-8^. Finally, at the complex GNAS locus at 20q13 the 11-marker moving window average BAF difference has a peak of 0.29 (below our cut-off) but the only SNPs in the CpG island, at 56,860,527 and 56,864,560 are at respectively 0.861 (*P*<10^-10^) and 0.586 (*P* =10^-5^). Clearly, not all known imprinted regions are detected as peaks of BAF difference in HIEC cells. This is expected in part because imprinting at a given locus is usually detected only in a limited number of tissues, and intestinal epithelium has not been specifically assessed for imprinting at many of these sites. Furthermore, most ICRs are only a few kb long, and many would not have heterozygous SNPs. This is the case for the ICR1 upstream of H19 as HIEC is homozygous from 1.973 to 2.000Mb, which includes this control region, and MEST at 7q34.

**Polyclonality of HIEC cells**

As mentioned above, polyclonal cell cultures should not yield peaks of BAF difference between anti-H3Ac ChIP and anti-H3M ChIP samples in domains of random inactivation, as occurs with the X chromosome. To check this, we calculated BAF differences for this chromosome in HIEC cells which are female-derived and reportedly polyclonal (results not shown). The highest peak was at 0.31, below what we consider a significant level. No regions where random monoallelic inactivation is known to occur (Chess, 2012; main text reference) yielded any peaks of BAF difference in HIEC cells, so we confirm the polyclonal nature of the cells.

Supplementary figure legends

Figure 1. Validation that BAF difference peaks reflect contiguous domains in HCT116

We reasoned that if BAF differences represented authentic differences between the two homologs, the material immunoprecitated by anti-Me should mainly be derived from one homolog, and that by anti-Ac mainly from the other throughout the length of high LD. Two regions under the long peak of BAF difference on chr1 (upper panel) were found for which HapMap data showed very high LD (r^2^ more than 0.9 throughout). The major allele frequency was plotted for anti-Ac (blue) and anti-Me (coral) for each SNP within the identified run of high LD. For every SNP, the anti-Ac ChIP yielded a higher BAF than the anti-Me ChIP, indicating perfect concordance between haplotype and immunoprecipitated material.

Figure 2. Validation that BAF difference peaks reflect contiguous domains in HIEC

See legend to SI Figure 1. Three regions of high LD on chr7 are shown. Again perfect concordance was observed between haplotype and immunoprecipitated material.

Figure 3 Validation that BAF difference peaks represent domains of monoallelic expression at SPRY2 (at 80.1Mb, chr13)

Genomic DNA and cDNA around rs504122 (C/T heterozygous in both HCT116 and HIEC) were amplified from both lines and sequenced. Upper panels, both alleles are expressed in HIEC, only the C in HCT116. Lower panel, BAF differences plotted for both cell lines, showing HCT116, but not HIEC, with BAF differences.
